# Synthesis, characterization and in vitro biocompatibility study of Au/TMC/Fe_3_O_4_ nanocomposites as a promising, nontoxic system for biomedical applications

**DOI:** 10.3762/bjnano.6.170

**Published:** 2015-08-03

**Authors:** Hanieh Shirazi, Maryam Daneshpour, Soheila Kashanian, Kobra Omidfar

**Affiliations:** 1 Endocrinology and Metabolism Research Center, Endocrinology and Metabolism Research Institute, Tehran University of Medical Sciences, Tehran, Iran; 2 Biosensor Research Center, Endocrinology and Metabolism Molecular-Cellular Sciences Institute, Tehran University of Medical Sciences, Tehran, Iran,; 3 Faculty of Chemistry, Nanoscience and Nanotechnology Research Center, Razi University, Kermanshah, Iran

**Keywords:** Au/polymer/Fe_3_O_4_ nanocomposites, Au nanoparticles, cell viability, magnetic nanoparticles, *N*-trimethyl chitosan

## Abstract

The unique properties and applications of iron oxide and Au nanoparticles have motivated researchers to synthesize and optimize a combined nanocomposite containing both. By using various polymers such as chitosan, some of the problems of classic core–shell structures (such as reduced saturation magnetization and thick coating) have been overcome. In the present study, chitosan and one of its well-known derivatives, *N*-trimethylchitosan (TMC), were applied to construct three-layer nanocomposites in an Au/polymer/Fe_3_O_4_ system. It was demonstrated that replacement of chitosan with TMC reasonably improved the properties of the final nanocomposites including their size, magnetic behavior and thermal stability. Moreover, the results of the MTT assay showed no significant cytotoxicity effect when the Au/TMC/Fe_3_O_4_ nanocomposites were applied in vitro. These TMC-containing magnetic nanoparticles are well-coated by Au nanoparticles and have good biocompatibility and can thus play the role of a platform or a label in various fields of application, especially the biomedical sciences and biosensors.

## Introduction

Nanotechnology is the science of the fabrication of novel materials, devices and systems by manipulating the molecular and atomic dimensions at the nanoscale. Nowadays, due to its widespread applications in various fields of science and technology (e.g., food industry, pharmaceutics, medical diagnosis, biotechnology, electronics, communication, transportation, energy, environment, aerospace, security [1–3]), nanotechnology is known as a cross-disciplinary science. Hence, most scientists are in agreement that this technology is not just a part of the future but will be a significant contributor to it.

To date, nanoparticles are the most widely applied, nanostructured material as they are considered to be a link between atomic structures and bulk material [4]. Many of the physicochemical properties of materials change as their size approaches the nanoscale, which can be explained by their high surface area to volume ratio and the quantum confinement effect [4–5].

Over the last decade, metal nanoparticles have attracted great scientific attention due to their diversity and wide-spread applications. By controlling the synthesis parameters for the development of metal nanoparticles of appropriate size, shape and structure, their applications can be expanded to various fields such as catalysis, electronics, photonics, sensors, microfluidic lateral flow devices, medical diagnosis, and related sciences [6–10].

One of the most important classes of metal nanoparticles are magnetic nanoparticles. Due to their unique properties, such as magnetic resonance momentum, super paramagnetism, super saturation, and having free electrons, magnetic nanoparticles have emerged as promising candidates for various medical and biological applications including magnetic resonance imaging (MRI) (as a contrast agent), smart drug delivery (as drug carriers), gene therapy, hyperthermia and tissue engineering, as well as the in the design of sensors and biosensors [11–17].

Although all nanoparticles containing a magnetic core are considered as magnetic nanoparticles, the most commonly used are iron oxide nanoparticles, which are mostly synthesized in the form of magnetite (Fe_3_O_4_) and maghemite (γ-Fe_2_O_3_). The increasing number of studies that report the successful use of Fe_3_O_4_ nanoparticles for industrial (e.g., as synthetic pigments or as catalyst), biomedical (in vivo and in vitro), environmental, and analytical applications, demonstrate their versatility. Since it is important that the Fe_3_O_4_ nanoparticles are stable under physiological conditions that are necessary for biomedical applications, it is crucial to use controllable synthesis conditions in order to obtain monodisperse, uniform nanoparticles with desired properties [18]. Despite their low toxicity, fine distribution, facile and low cost synthesis process, and ease of surface modification, biological and medical applications using uncoated iron oxide nanoparticles are limited because of their tendency to aggregate and oxidize [9,19]. Covering their surface with organic molecules (e.g., biodegradable polymers, noble metals such as gold, or oxide layers such as silica or alumina) not only prevents their aggregation due to the change in surface charge, but also protects them from oxidation [12,20–21]. Additionally, proper surface coverage increases the stability and half-life of the magnetic nanoparticles when used in biological environments due to their delayed clearance through the renal system and deterrence to opsonization by the immune system [22–23]. In addition, by means of various coating materials, it is possible to improve their biomedical properties by inhibiting their undesirable interactions with cells and proteins [12]. Biodegradable polymers that are generally used for coating magnetic nanoparticles include poly(ethylene glycol) (PEG), poly(vinyl alcohol) (PVA), polyethyleneimine (PEI), poly(D,L-lactide), poly(lactic acid), poly(D,L-glycolide), poly(lactide-*co*-glycolide), polycyanoacrylate, alginate, gelatin, and chitosan [19,23–24].

Among these polymers, chitosan has received significant commercial attention due to its outstanding properties such as nontoxicity, biocompatibility, biodegradability, adsorption, and its ability to form films and to chelate metal ions [25–26]. This fiber-like polymer is made of ß(1→4)-linked *N*-acetyl-D-glucosamine and derives from chitin. After cellulose, chitosan is known as the second most abundant organic compound in nature. A major disadvantage of using chitosan is its poor solubility at physiological pH, whereas it is soluble and active only in an acidic environment in its protonated form [25,27]. In contrast, *N*-trimethylchitosan chloride (TMC), a partially quaternized chitosan derivative, shows good water solubility over a wide pH range [28].

In this study, magnetic nanoparticles (Fe_3_O_4_) are coated with either chitosan or TMC. Given that these polymers provide an adsorbent network on the surface of the Fe_3_O_4_ nanoparticles, it was decided to additionally assemble Au nanoparticles onto the polymer-coated magnetic particles, resulting in the development of novel nanocomposites. The final diameter of the resulting nanocomposite was less than 100 nm, which is ideal for avoiding rapid clearance by the reticuloendothelial system [18]. These nanocomposites showed suitable magnetic potential, and due to the many remarkable properties of Au nanoparticles (e.g., high chemical stability, resistance to oxidation, and excellent biocompatibility), they can be utilized as catalysts, labels, and as a protective substrate, especially for immobilization of biomolecules in various fields of modern science [29–30]. Au nanoparticles are extensively used in the design and construction of fuel cells and many types of sensors (e.g., electrochemical, analytical, colorimetrical and fluorescent) due to their unique catalytic capability and extraordinary sensitivity and selectivity [29,31]. Au nanoparticles are typically synthesized in colloidal form in aqueous solution and their properties and applications significantly depend upon their size and shape. Generally, Au nanoparticles are synthesized by reduction of chloroauric acid (HAuCl_4_). Various reducing agents are employed to produce Au^0^ atoms, which act as nucleation centers [32]. By controlling the various synthesis conditions, such as the reducer used, thermal conditions, and reaction steps, it is possible to prepare Au nanoparticles of different sizes and shapes that will enhance their efficacy and usage in different applications [33].

It is expected that the nanocomposites developed herein will have advantages over other hybrid Fe_3_O_4_ and gold core–shell structures. This is particularly anticipated in the fields of electrochemical sensors and biosensors, where Au nanoparticles play a fundamental role as labels or platforms for immobilization. In this respect, our results suggest that enhanced signal amplification and increased magnetic separation efficiency are likely. In addition, the nontoxic nature of this nanocomposite (which was explored by a 3-(4,5-dimethylthiazol-2-yl)-2,5-diphenyltetrazolium bromide (MTT) assay), facilitates its application in various in vitro and in vivo settings.

This work focuses on the preparation process of the novel, gold-coated, iron oxide, chitosan/TMC-containing, magnetic nanoparticles and their respective characterization using transmission electron microscopy (TEM), ultraviolet–visible absorption spectroscopy (UV–vis), X-ray diffraction spectroscopy (XRD), Fourier transform infrared spectroscopy (FTIR), a vibrating sample magnetometer (VSM), energy dispersive X-ray diffraction (EDXD), dynamic light scattering (DLS), and thermogravimetric analysis (TGA).

## Results and Discussion

In this study we successfully designed, synthesized, and compared two hybrid magnetic–gold nanocomposites by applying chitosan and one of its well-known derivatives, TMC. This system not only takes advantage of the combined useful properties of both Fe_3_O_4_ and Au nanoparticles, but also has promising characteristics that resolve many of the previously reported shortcomings of classic core–shell structures [34].

### Fe_3_O_4_ nanoparticles as the magnetic core

Among the different methods for iron oxide nanoparticles synthesis, co-precipitation was chosen due to its simplicity, low reaction temperature, short synthesis time, very high yield, and biocompatible product [20]. As previously proposed [19], the molar ratio of Fe^+2^/Fe^+3^ = 0.5 and pH 11–12 were applied in this study to achieve the optimum results presented by the reaction:

2FeCl_3_ + FeCl_2_ + 8NaOH → Fe_3_O_4_(s) + 4H_2_O + 8NaCl.

**Figure 1:**
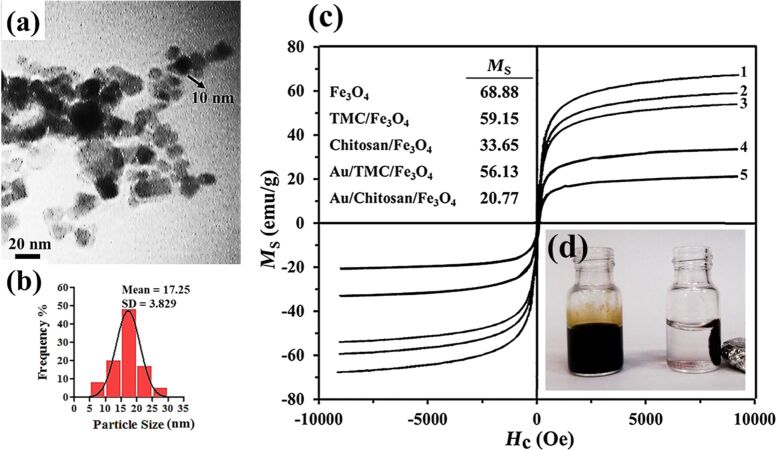
(a) TEM image of uncoated Fe_3_O_4_ nanoparticles and their (b) corresponding particle size distribution. (c) Hysteresis loop of the synthesized magnetic nanoparticles: (1) Fe_3_O_4_, (2) TMC/Fe_3_O_4_, (3) Au/TMC/Fe_3_O_4_, (4) chitosan/Fe_3_O_4_ and (5) Au/chitosan/Fe_3_O_4_. (d) Iron oxide suspensions with (right) and without (left) an external magnetic field.

As the TEM image in Figure 1a shows, the typical uncoated Fe_3_O_4_ nanoparticles had an average diameter of 10 nm and were monodisperse and partially aggregated because of their magnetic nature.

From the DLS analysis (Figure 1b and Table 1), the average diameter of the iron oxide nanoparticles was found to be 17 ± 3.83 nm. Moreover, the saturated magnetization of the uncoated Fe_3_O_4_ nanoparticles was found to be 68.88 emu g^−1^ (Figure 1c), which was lower than the reported VSM value for bulk magnetic materials (92 emu g^−1^). This could be explained by the fact that the magnetic field for nanoparticles of diameter less than 15 nm decreases as the particle size decreases [35]. Such an acceptable saturated magnetization facilitates the subsequent coating step.

**Table 1:** Size evaluation via TEM, XRD, DLS and zeta potential analysis of the synthesized nanoparticles.

Nanoparticle structure	Diameter (nm)			Zeta potential (mV)
	TEM	XRD	DLS	

Au	10 ± 2	9	10.59 ± 3.83	ND^a^
Fe_3_O_4_	10 ± 1	10	17.25 ± 3.08	−42.7 ± 0.82
chitosan/Fe_3_O_4_	25 ± 2	26	68.09 ± 5.24	+23.6 ± 1.51
TMC/Fe_3_O_4_	12 ± 1	13	21.50 ± 3.43	+41.5 ± 3.51
Au/chitosan/Fe_3_O_4_	40 ± 3	42	99.63 ± 7.49	−6.2 ± 0.81
Au/TMC/Fe_3_O_4_	20 ± 2	23	33.80 ± 3.44	−31.6 ± 1.61

^a^Data not determined.

### Au nanoparticles

**Figure 2:**
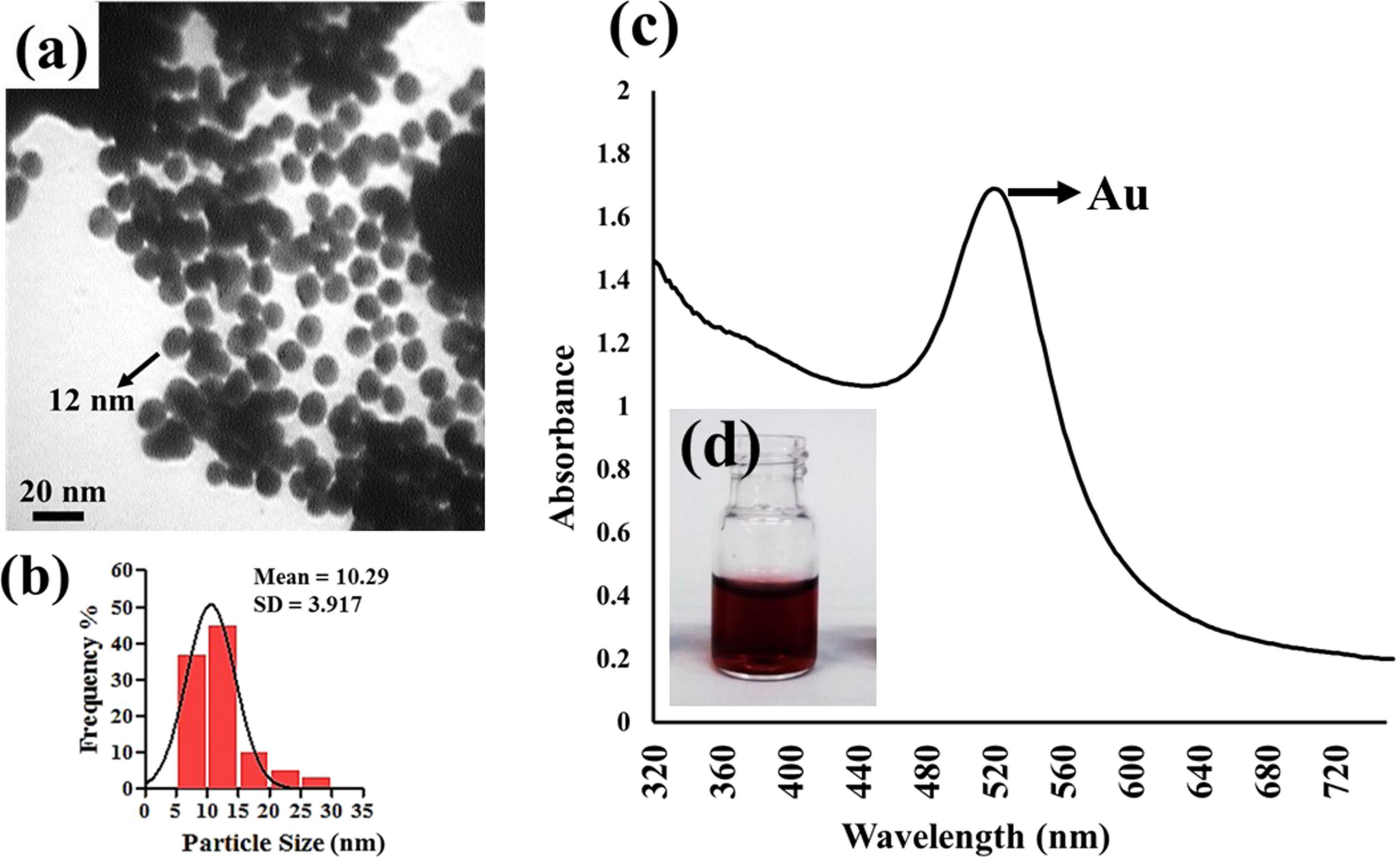
(a) TEM image of Au nanoparticles along with (b) their corresponding particle size distribution. (c) UV–vis absorption spectrum of synthesized Au nanoparticles. (d) Image of a freshly prepared, ruby-red, colloidal, Au nanoparticle solution.

There is a variety of reported methods for the synthesis of colloidal Au nanoparticles all of which are generally based on the reduction of Au salts such as HAuCl_4_ [31]. In this study, D-glucose, as the reducing agent, was oxidized to gluconic acid, which then forms a very thin layer on the surface of the reduced Au nanoparticles. This layer acted to further stabilize the Au nanoparticles via interactions with hydroxyl groups [33,36]. The use of an alkaline solution (NaOH) stimulates the opening of the glucose ring, which facilitates the Au^3+^ reduction [36]. Through regulation of the reaction conditions and parameters, the final Au nanoparticles with a desirable diameter of 10 ± 2 nm (Figure 2a,b) and negative surface charge were obtained. The UV–vis absorption spectrum of a ruby-red colloidal solution of Au nanoparticles is presented in Figure 2c. Due to the excitation of surface plasmon vibrations, a strong peak can be observed near 520 nm. This peak corroborates the presence of Au nanoparticles and their spherical morphology.

### Chitosan/Fe_3_O_4_ and TMC/Fe_3_O_4_ nanoparticles

**Figure 3:**
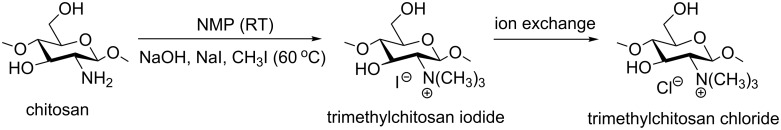
TMC synthesis procedure.

First, TMC with a degree of quaternization DQ of about 36% was synthesized as explained above. This process is schematically represented in Figure 3. Next, chitosan and TMC were introduced to the magnetic core in the presence of glutaraldehyde as a cross-linker.

**Figure 4:**
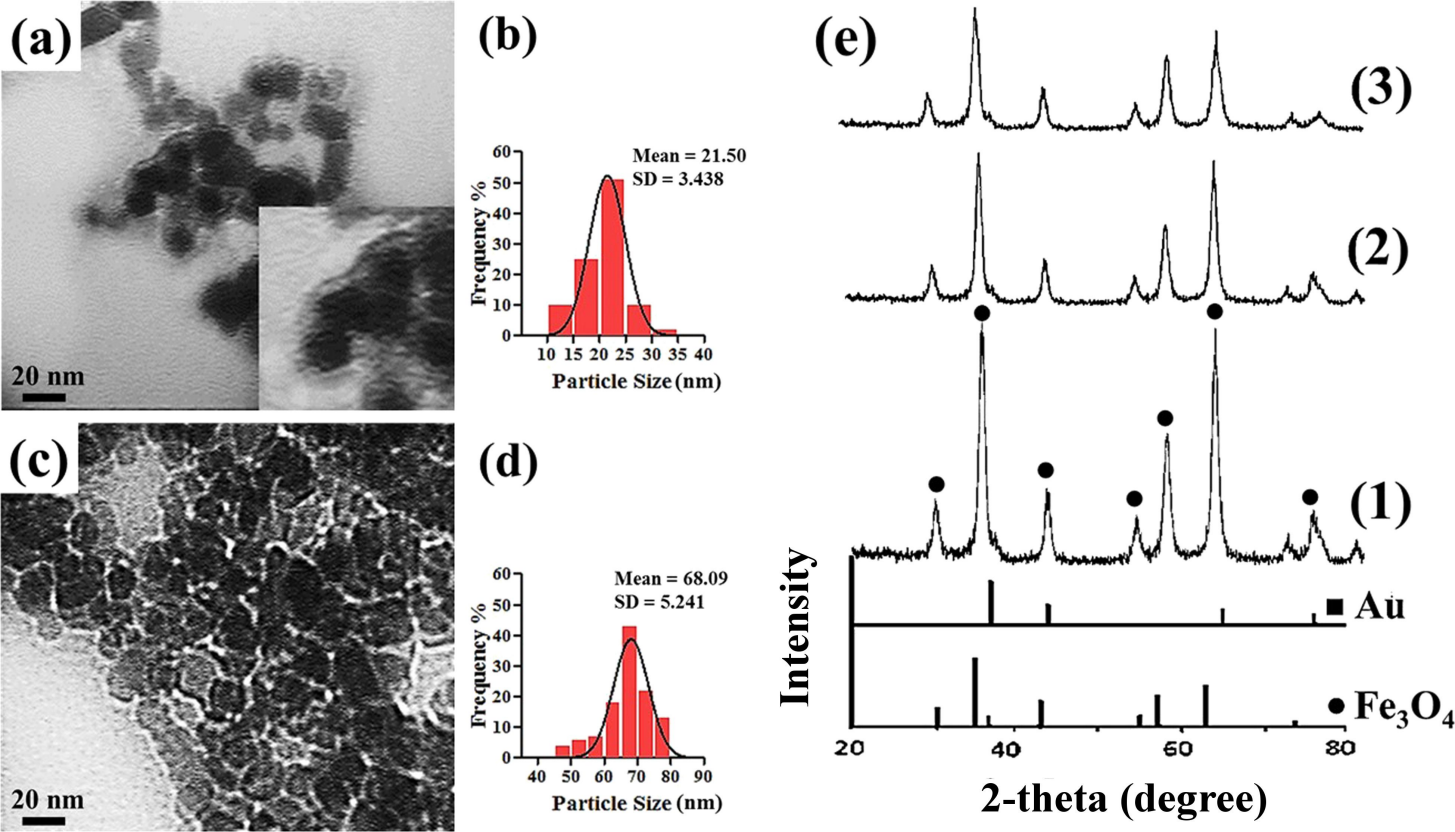
TEM images of (a) TMC/Fe_3_O_4_ and (c) chitosan/Fe_3_O_4_ nanoparticles and their particle size distributions (b and d, respectively). (e) XRD diffractograms of (1) uncoated Fe_3_O_4_ (2) TMC/Fe_3_O_4_ and (3) chitosan/Fe_3_O_4_.

According to the many reports on materials used for coating or encapsulating iron oxide nanoparticles, using chitosan results in particles with a rather broad distribution (20–100 nm) [37]. According to the TEM images, the chitosan-coated magnetic nanoparticles had a diameter of about 25 nm, which is reasonable compared to similar reported nanoparticles. The TEM results and particle size distribution of the polymer-coated Fe_3_O_4_ nanoparticles (Figure 4a–d) indicated an interesting effect with respect to the exchange of TMC for chitosan: The TMC-coated nanoparticles exhibited a smaller diameter as compared to the chitosan-coated nanoparticles. No significant nanoparticle agglomeration was observed upon introduction of the polymers. The core–shell structure of the TMC/Fe_3_O_4_ nanoparticles is clearly visible in Figure 4a. The Fe_3_O_4_ nanoparticles are encapsulated by a contrasting layer of TMC, which inhibited aggregation due to the change in surface charge. In contrast, the polymer shell was not visibly recognizable in the TEM image of the chitosan/Fe_3_O_4_ nanoparticles (Figure 4c). It seemed that the magnetic nanoparticles were rather embedded in a chitosan matrix.

According to the hysteresis curves (Figure 1c), the saturation magnetization (*M*_s_) of TMC/Fe_3_O_4_ nanoparticles was 59.15 emu/g, which was notably higher than 33.65 emu/g found for the chitosan/Fe_3_O_4_ nanoparticles. Both of these nanoparticles showed a smaller *M*_s_ value as compared to uncoated magnetic nanoparticles, which provides evidence of the formation of a polymer layer around the Fe_3_O_4_ nanoparticles. The XRD results (Figure 4e) illustrated seven intense peaks at about 30.03°, 35.27°, 43.10°, 53.64°, 57.16°, 62.21° and 73.91° that correspond to the following Miller indices: (220), (311), (400), (422), (511), (440), and (533), indicating a spinel structure for Fe_3_O_4_ [38]. The average nanoparticle diameter was determined using the XRD data and the Debye–Scherrer equation, which were in agreement with the TEM results. According to the XRD results, the apparent similarity between the diffractograms of the uncoated Fe_3_O_4_ nanoparticles and the polymer/Fe_3_O_4_ nanoparticles in Figure 4 demonstrates that the coating process did not change the phase of the Fe_3_O_4_ and only resulted in a slight decrease in peak intensity. The nanoparticle diameters as evaluated by XRD and DLS analysis are summarized in Table 1.

The study of the surface properties of nanoparticles, especially their surface charge in different physicochemical and physiological conditions, is important to understanding their interactions with other molecules in in vivo and in vitro microenvironments. Hence, the surface charge of the nanoparticles was investigated by means of zeta potential analysis. As shown in Table 1, the uncoated Fe_3_O_4_ nanoparticle surface charge was about −42.7, which changed to +41.5 and +23.6 after coating with TMC and chitosan, respectively. This was well expected because of the positive amine groups of the polymer backbone.

**Figure 5:**
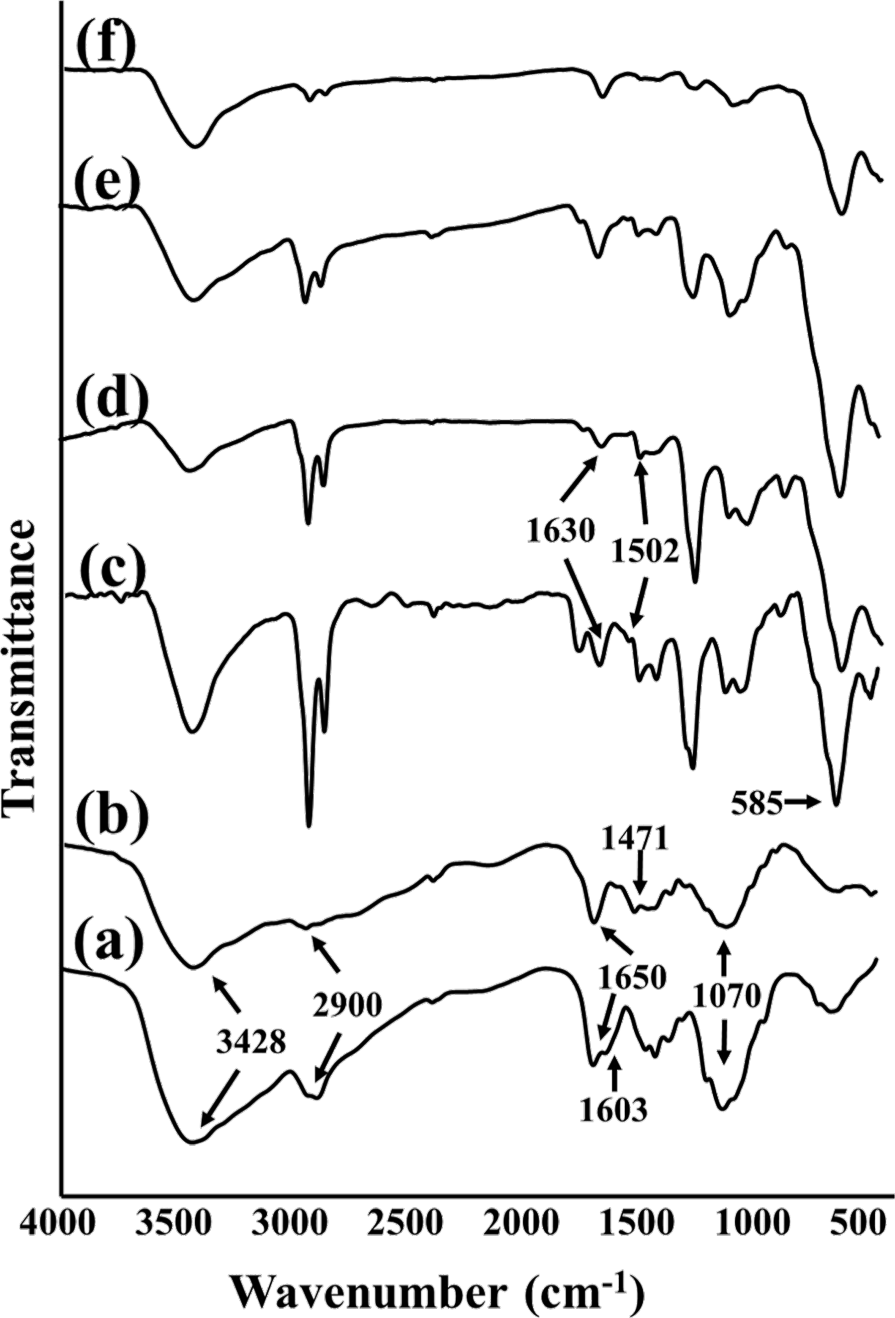
FTIR spectra of (a) chitosan, (b) TMC, (c) chitosan/Fe_3_O_4_, (d) TMC/Fe_3_O_4_, (e) Au/chitosan/Fe_3_O_4_, and (f) Au/TMC/Fe_3_O_4_.

Decisive evidence of the successful synthesis of polymer-coated Fe_3_O_4_ nanoparticles was obtained from FTIR analysis. This technique was used to understand the nature of the bonds and functional groups of the molecules and how they were altered during the synthesis processes. The FTIR spectra of chitosan and TMC are given in Figure 5a,b. Four characteristic absorbance bands of these familiar polymers are clearly discernable. As can be seen, the overlapping of the O–H and N–H stretching vibrations resulted in a broad peak at 3428 cm^−1^. The distinct peak at 2900 cm^−1^ is assigned to the C–H bond. The absorption near 1650 cm^−1^ is ascribed to the N–H bending vibration. The last major peak is due to the C–O–C stretching vibration, which is observable near 1070 cm^−1^. However, there are two important differences between the patterns of TMC and chitosan: for TMC, there is a peak at about 1471 cm^−1^ related to the C–H asymmetrical stretching mode present in methyl groups, which was not detected in the chitosan spectrum. Also, near 1603 cm^−1^ in the FTIR spectrum of chitosan, the N–H angular deformation peak of the amine groups was reduced in the TMC spectrum. This peak is believed to be overlaid by the carbonyl stretching of the acetamide moiety [39]. Figure 5c,d illustrates the FTIR spectra of polymer/Fe_3_O_4_ nanoparticles. The strong peak that appears at about 585 cm^−1^ is an indicator of Fe–O. A notable peak near 1502 cm^−1^ in the polymer/Fe_3_O_4_ spectra correlates with the shifted N–H bond. This is in addition to a new peak at about 1630 cm^−1^ which is supposed to be the result of the Schiff base formation due to the interaction between the N–H groups of the polymers and the CHO group of glutaraldehyde (which was used as the cross-linker during the polymer/Fe_3_O_4_ nanoparticle synthesis) [40].

### Au/chitosan/Fe_3_O_4_ and Au/TMC/Fe_3_O_4_ nanocomposites

Because of the numerous advantages of nanocomposites containing both Fe_3_O_4_ and Au, the synthesis of such hybrid structures has become one of the most quickly advancing research areas in recent decades. Wu et al. have comprehensively reviewed and compared many different nanocomposite systems [19]. There are also a few studies illustrating the application of chitosan as a medium layer on a magnetic core for Au functionalization. As was reported, the electrostatic adsorption of negatively charged Au nanoparticles onto a positively charged chitosan layer resulted in nanocomposites rather similar to our Au/chitosan/Fe_3_O_4_ nanoparticles [38,41]. These studies achieved a final nanocomposite diameter in size range of 100–300 nm, which are significantly larger than our Au/chitosan/Fe_3_O_4_ nanoparticles. Such dimensions might limit their applications in in vivo settings. In the following, the effects of using TMC in the synthesis of such nanocomposites are presented and compared to the Au/chitosan/Fe_3_O_4_ nanoparticles.

**Figure 6:**
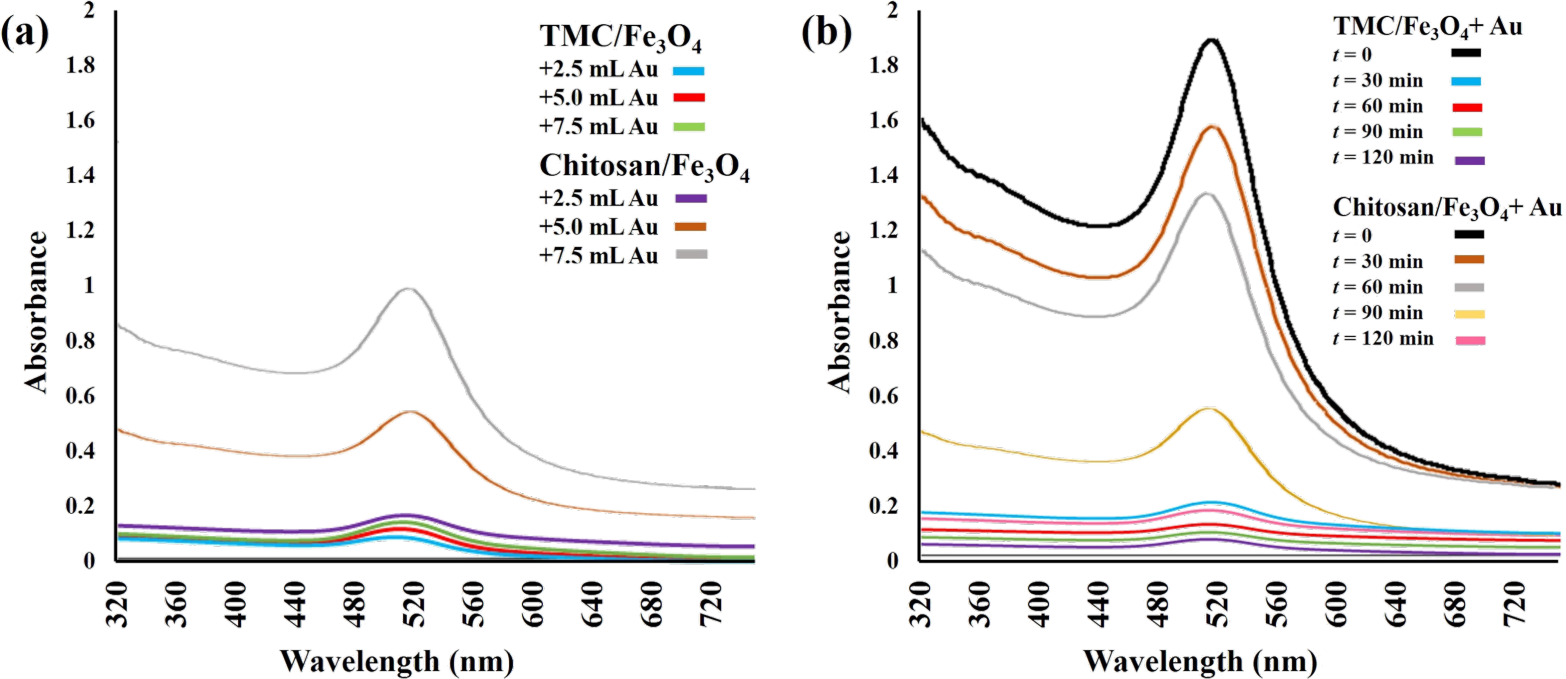
UV–vis spectroscopy of Au nanoparticles as a function of (a) Au nanoparticle concentration and (b) reaction time.

The UV–vis adsorption analysis indicated that the TMC-containing nanoparticles absorbed nearly all the Au nanoparticles in all three samples. This also caused the pinkish color of the solutions to disappear. However, the spectra showed the obvious presence of Au nanoparticles in the case of the magnetic chitosan nanoparticles, which was due to their limited capacity to adsorb the Au nanoparticles (Figure 6a). The results of the comparison of Au adsorption by polymer/Fe_3_O_4_ nanoparticles as a function of time are shown in Figure 6b. According to the spectra, it seems that all the Au nanoparticles have been absorbed onto the TMC/Fe_3_O_4_ nanoparticles within the first 15 min. In contrast, the absorbance value for chitosan/Fe_3_O_4_ gradually decreased after two hours of incubation. This effect was also demonstrated via other experiments and could be the result of the difference in molecular composition of chitosan and TMC.

**Figure 7:**
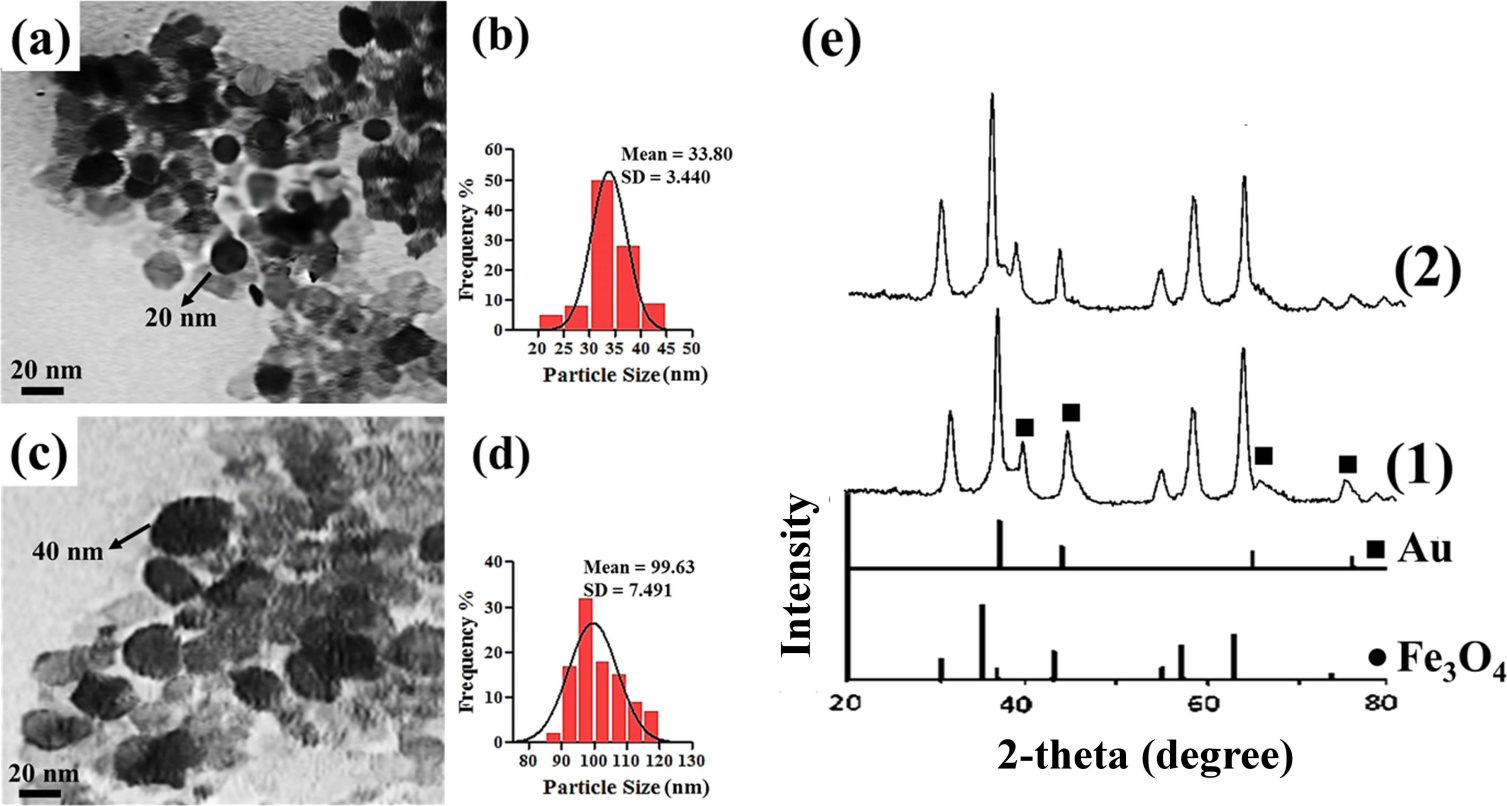
TEM images of (a) Au/TMC/Fe_3_O_4_ and (c) Au/chitosan/Fe_3_O_4_ with respective particle size distributions (b and d, respectively). (e) XRD patterns of (1) Au/TMC/Fe_3_O_4_ and (2) Au/chitosan/Fe_3_O_4_.

TEM images of Au/polymer/Fe_3_O_4_ nanocomposites are shown in Figure 7a,c. The darker, spherical nanoparticles (with a mean diameter of about 20 nm for Au/TMC/Fe_3_O_4_ nanoparticles and 40 nm for Au/chitosan/Fe_3_O_4_ nanoparticles) indicate successful attachment of Au nanoparticles onto the polymer-covered Fe_3_O_4_ nanoparticles. By comparison, it can be seen that a greater amount of Au was attached onto the TMC-coated magnetic nanoparticles.

Due to the assembly of the Au nanoparticles onto the polymer/Fe_3_O_4_ nanoparticle surface, four new peaks appeared in the XRD spectra at (111), (200), (220) and (311), which indicated the presence of Au nanoparticles in the final nanocomposites [42]. However the diffraction peaks of iron oxide seemed to be weakened compared to those of uncoated Fe_3_O_4_. This can be explained by the higher electron configuration of Au, which is known as the heavy atom effect [42]. The relatively broad nature the Bragg reflections also indicated that the synthesized particles were on the nanoscale. The diameter of the final products were evaluated from XRD data to be 23 nm and 42 nm for Au/TMC/Fe_3_O_4_ and Au/chitosan/Fe_3_O_4_ systems, respectively, which were in good agreement with the TEM results. It seems that the Au peaks of the Au/TMC/Fe_3_O_4_ nanoparticles are more intense, suggesting a higher concentration of Au in these nanocomposites.

Through DLS analysis (Figure 7b,d, Table 1), the diameters of the nanoparticles were generally found to be greater than those obtained by TEM. The poor accuracy of the DLS technique for nanoparticles size measurements, especially in magnetic samples, has been previously reported and could be due to the high tendency of the nanoparticles to aggregate [43–44]. To attempt to overcome this problem, ultrasonic agitation was applied for about 10 min before the measurements. Nevertheless, it seems that the true nanoparticle diameter could not be determined using the DLS technique. Despite this, the DLS results revealed a narrow size distribution and also showed the reasonable increase in nanoparticle diameter as the polymer layers and Au were added onto the magnetic core.

The attachment of Au onto the polymer/Fe_3_O_4_ nanoparticles also influenced the surface charge of the resulting nanoparticles. The results of the zeta potential analysis in Table 1 show that the surface charge of the final products are −31.6 for Au/TMC/Fe_3_O_4_ nanoparticles and −6.2 for Au/chitosan/Fe_3_O_4_ nanoparticles due to the negative charge of Au anions. As expected, the higher concentration of Au in the TMC-grafted magnetic nanoparticles resulted in a more negative value of the final nanocomposites as compared to the chitosan-containing nanoparticles.

**Figure 8:**
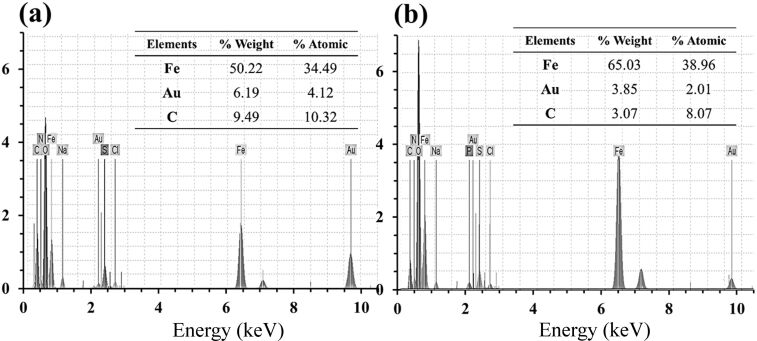
EDXD spectra of (a) Au/TMC/Fe_3_O_4_ and (b) Au/chitosan/Fe_3_O_4_.

In order to further study the chemical composition, the final nanocomposites were subjected to EDXD analysis. According to the spectra in Figure 8, the existence of Fe, Au and O were verified as well as other elements such as H, N and C (which are due to the chitosan and TMC polymers). As can be seen, the concentration of Au in TMC-containing nanocomposites is higher than those with chitosan, which is in agreement with the results of other analyses. In addition, higher value of C in the TMC-contained sample could be related to the methyl groups from the chitosan molecules from the TMC synthesis.

Figure 1e shows that the measured saturated magnetization for Au/chitosan/Fe_3_O_4_ and Au/TMC/Fe_3_O_4_ nanoparticles was 20.77 and 56.13 emu/g, respectively. While all synthesized nanoparticles exhibited good magnetic properties, even in the presence of polymers and Au nanoparticles, the addition of a diamagnetic layer of chitosan caused a more significant reduction in the magnetic behavior as compared to TMC. The zero remanence and coercivity values indicated the superparamagnetic nature of these nanoparticles. In accordance with the direct relation between crystallinity and magnetization of magnetic nanoparticles, these results confirm the crystallinity of the nanoparticle systems [35]. Given that the applications of such nanoparticles are based on their sufficient magnetic properties, the use of TMC (with better magnetic properties as compared to the classical chitosan system) is recommended, especially in biomedical applications.

### Thermogravimetric analysis (TGA)

**Figure 9:**
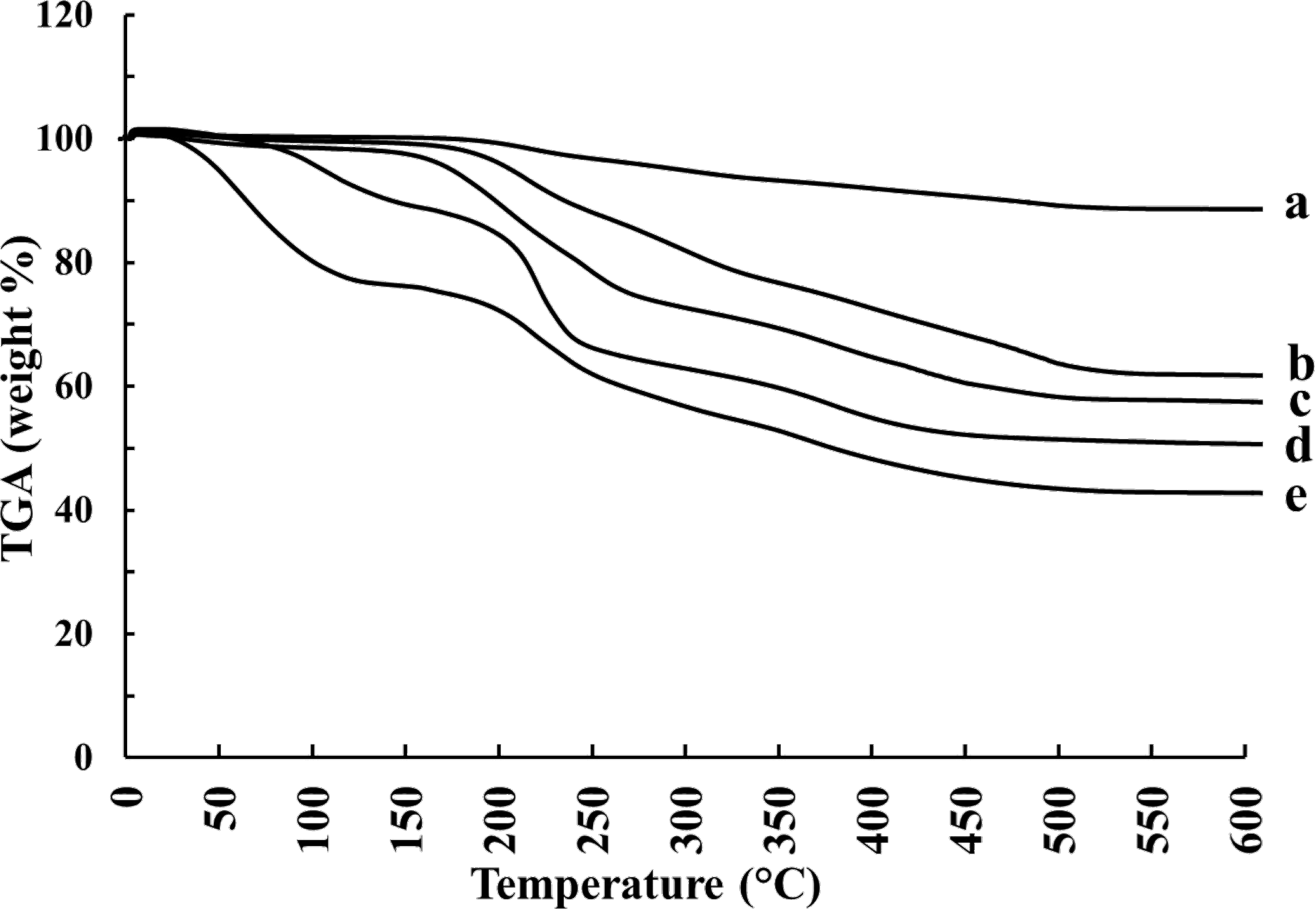
Thermal degradation curves of synthesized nanoparticles: (a) Fe_3_O_4_, (b) Au/TMC/Fe_3_O_4_, (c) TMC/Fe_3_O_4_, (d) Au/chitosan/Fe_3_O_4_, and (e) chitosan/Fe_3_O_4_.

During the TGA measurement (Figure 9), the first major weight loss occurred at 25–300 °C, which is related to the removal of intramolecular, chemisorbed and physisorbed water [45]. As previously reported, the second weight loss step arises from the decomposition of organic compounds, such as the polymers, which starts at 250 °C. The amount of coated polymer was evaluated by comparing the TGA curves of uncoated Fe_3_O_4_ and polymer/Fe_3_O_4_ nanoparticles [35]. The third weight loss step can be ascribed to the complete conversion of the materials into oxides [46]. In this way, the weight percentages of chitosan and TMC in the chitosan/Fe_3_O_4_ and TMC/Fe_3_O_4_ systems were found to be 47% and 32%, respectively. The TGA results indicate that Au-containing nanocomposites lost less weight during the second step. The reason for this could be due to the interaction of Au nanoparticles with polymers. The remaining weight of Au-containing nanocomposites is more than that of polymer/Fe_3_O_4_ nanoparticles, which is likely due to the relatively heavy weight of Au nanoparticles.

### Cell viability assay

**Figure 10:**
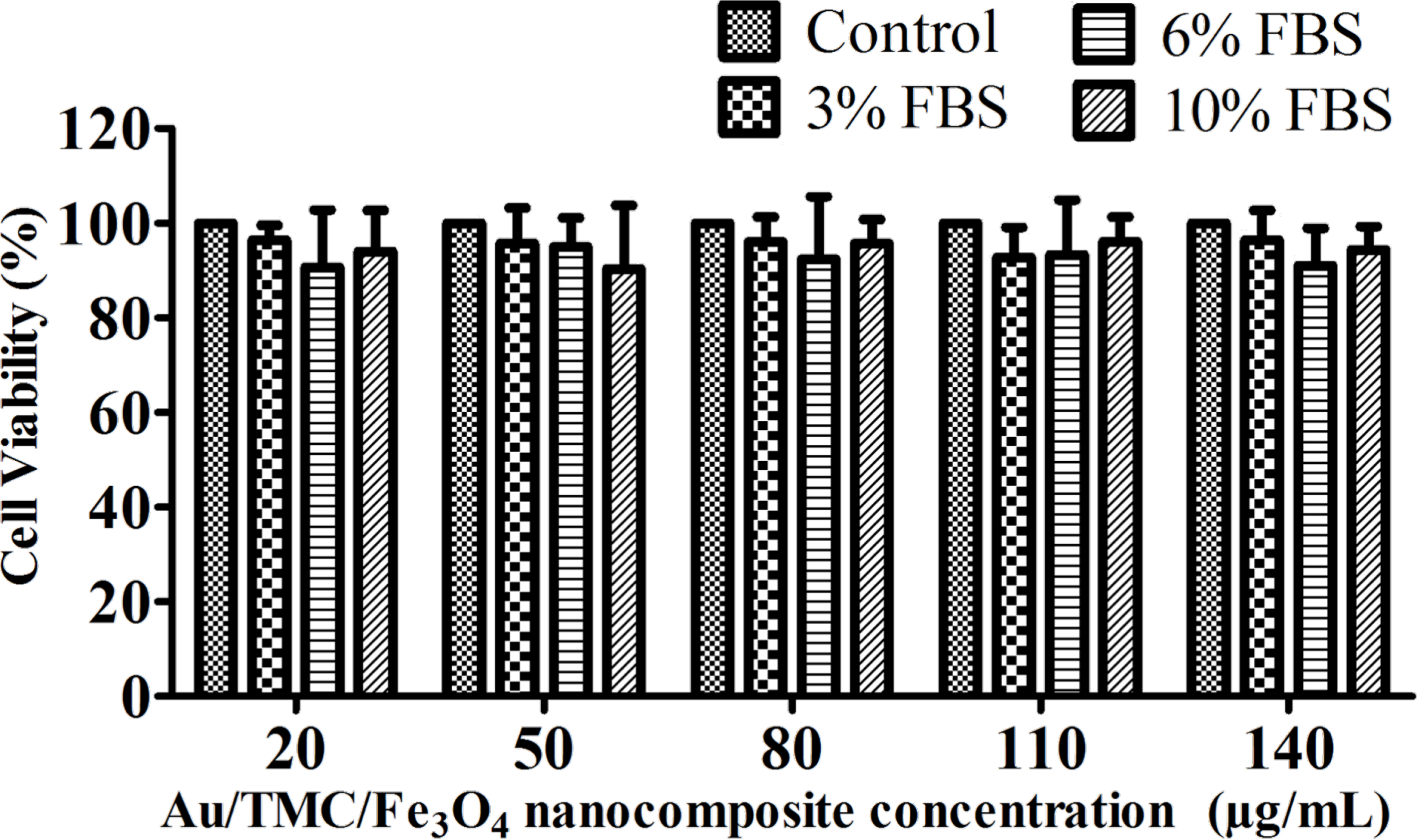
The effect of different concentrations of Au/TMC/Fe_3_O_4_ nanoparticles on cell viability as assessed by MTT assay.

One of the most important factors for employing nanomaterials in biomedical applications is related to their safety and biocompatibility. The interaction of nanoparticles with cell membranes, their ingestion by cells, and their intracellular storage may have negative effects on the cells, regardless of the toxicity of the particles and their subsequent functionality. Numerous studies have reported magnetic-nanoparticle-induced cell death to various degrees and ascribe this mainly to the production of reactive oxygen species (ROS) [47–48]. However, the coated nanoparticles have shown improved stability and biocompatibility in in vivo and in vitro assays [49]. In this study, the effects of Au/TMC/Fe_3_O_4_ nanocomposites on cell viability were assessed using the MTT assay. The assay was based on the reduction of the dye MTT to formazan crystals (an insoluble, intracellular, blue product) by cellular dehydrogenases. The MTT results demonstrated that no significant decrease in cell viability occurred in presence of different concentrations of our synthesized nanocomposite. The medium concentration also had no remarkable effect: the cell viability remained between 90–96% after 48 h, and there was no considerable difference between the treated and control cells (Figure 10). Therefore, the proposed nanostructure is considered to be biocompatible and nontoxic to the cells.

## Conclusion

In summary, this study reports the preparation of two nanocomposites using a three step procedure. The monodisperse, uniform, Fe_3_O_4_ nanoparticles were synthesized through a co-precipitation method resulting in a diameter of about 10 nm, followed by surface modification by a chitosan or TMC coating. The successful coating process was confirmed by FTIR. These nanocomplexes were then exposed to Au nanoparticles, which assembled on the surface to form the final nanoparticles. The size, magnetic behavior, chemical composition, thermal stability and surface characteristics of all nanoparticles were evaluated via a variety of techniques such as TEM, XRD, EDX, DLS, VSM, TGA, and FTIR. The development of novel three-layer particles with a core–shell–shell structure, proper size range, and good saturated magnetization were confirmed which also showed a low concentration of biocompatible polymers. The final nanocomposites were less than 100 nm in diameter, which is considered ideal to avoid rapid clearance by the reticuloendothelial system.

Because it is well characterized and nontoxic, chitosan has gained significant interest in bio-nano applications. The methylation of chitosan results in the formation of an excellent polymer (TMC) with improved properties. As demonstrated in this work, TMC covers the magnetic core as a very thin, positive layer and provides welcoming conditions for Au assembly. Although both of the polymer-containing nanocomposites have great potential for applications in nanotechnology, this study shows an incredible improvement in the nanoparticle properties by using the quaternized derivative of chitosan, TMC. Our results demonstrate that TMC has an even better efficacy than chitosan with respect to nanocomposite construction, suggesting that replacement with TMC might overcome some of the limitations of chitosan (and even other polymers).

Considering its biocompatible and nontoxic properties, the Au/TMC/Fe_3_O_4_ nanocomposite developed herein could be promisingly employed as a multifunctional platform for biomedical applications. The efficacy of these TMC- and Au-containing magnetic nanostructures could benefit applications such as electrochemical labels, sensory probes, electronic conductors, therapeutic agents, organic photovoltaics, drug delivery in biological and medical applications, and catalysis due to their combined magnetic and optoelectronic properties.

## Experimental

### Reagents and solutions

Chloroauric acid (HAuCl_4_), sodium dodecyl sulfate (SDS), sodium azide, low molecular weight chitosan, MTT reagent, RPMI1640 cell culture medium, fetal bovine serum (FBS), and dialysis tubing with a molecular cutoff of 12000 Da were purchased from Sigma (UK). T47D human ductal breast epithelial tumor cell line was obtained from American Type Culture Collection (ATCC) (USA). FeCl_3_·6H_2_O (99.0%), FeCl_2_·4H_2_O (99.0%), NaOH, and acetic acid were purchased from Acros Organics (USA). Analytical HCl, NaCl, D-glucose, glutaraldehyde, *N*-methylpyrolidone (NMP), iodomethane, sodium hydroxide, sodium iodide, and acetone were purchased from Merck (Darmstadt, Germany). Deionized water was used in all experiments.

### Synthesis of Fe_3_O_4_ magnetic nanoparticles

NaOH was used as the reduction agent for the synthesis of iron oxide nanoparticles via co-precipitation. 2.365 g of FeCl_3_·6H_2_O and 0.99 g of FeCl_2_·4H_2_O were added to 100 mL of distilled and deoxygenated water and stirred about 30 min in the presence of N_2_. After adding 50 mL of NaOH (2 M) to the mixture, the pH was adjusted to about 12. The color of the mixture turned brown and then black after stirring for several minutes, which was the sign of the formation of the magnetic nanoparticles. The nanoparticles were cooled to rt and washed multiple times. For storage, tetramethylammonium hydroxide (TMAH) (0.1 M) was added to the solution [50].

### Synthesis of Au nanoparticles

D-glucose was used as the reducer for the preparation of Au nanoparticles with an optimum size of 10 ± 2 nm. First, 18 mL of HAuCl_4_ (6 × 10^−4^ M) was mixed with 2 mL of D-glucose (1 M) and the prepared solution was stirred at 60 °C. Next, 40 µL of NaOH (1 M) was added while the reaction mixture was stirring. After about 10 s, the then pinkish-red solution containing the Au nanoparticles was cooled down and the pH was adjusted to 8.5. Lastly, sodium azide (0.01%) was added and the final solution was stored in a dark bottle at 4 °C [33].

### Synthesis of *N*-trimethylchitosan

For synthesis of *N*-trimethylchitosan, 1 g of low molecular weight chitosan and 50 mL of NMP were mixed and stirred for 12 h at rt. Then, 8 mL of sodium hydroxide (15%) and 3 g of sodium iodide were added to the mixture which was stirred for 15 min at 60 °C. Later, 8 mL of iodomethane (as the source of methyl groups) were added in three steps every three hours and the solution was stirred for 24 h at 60 °C. The primary polymer was precipitated by means of an appropriate amount of acetone and then solved into NaCl (15%). The resulting suspension was dialyzed against deionized water for three days and finally freeze-dried, producing a pale yellow, TMC powder [28,51–52].

### Polymer-coated Fe_3_O_4_ nanoparticles

First, 0.2 g of the previously sonicated iron oxide nanoparticles were dispersed in a 25.5 mL solution containing NaCl (0.5 M) and SDS (0.025 M). Given the molar ratio of polymer and Fe_3_O_4_ (1:1), an appropriate amount of chitosan or TMC (in 2% acetic acid) solution was added to the suspension and the mixture was sonicated at 20 °C for 1 h. Then, 2.75 mL of glutaraldehyde (25%) was added as the cross-linker and the suspension was stirred at 50 °C for about 5 h. Lastly, the products were washed several times using external magnets and the process was completed by adding sodium azide (0.01%) and drying the final coated nanoparticles in the oven. The resulting black powder was stored at 4 °C in a dark bottle [38].

### Au nanoparticle-covered magnetic nanoparticles

0.2 g of the polymer-coated Fe_3_O_4_ nanoparticles were exposed to 25 mL of the previously synthesized, Au nanoparticle, colloid solution for about 2 h under continuous stirring conditions. The Au nanoparticles were assumed to attach to the polymer/Fe_3_O_4_ complex via electrostatic interaction, which was confirmed by the discoloration of the mixture. After washing with deionized water and adding sodium azide (0.01%) and phosphate buffered saline (PBS) (pH 7.2, 0.01 M), the product was stored at 4 °C in a dark bottle [38].

### UV analysis of Au nanoparticles

In this study, two modes of analysis were performed to compare the concentration of the Au nanoparticles and the rate at which they were attached onto the surface of the polymer/Fe_3_O_4_ complex. For the first one, 3 portions of equal amounts of polymer/Fe_3_O_4_ nanoparticles (20 mg) were subjected to different volumes of Au nanoparticle solutions (2.5, 5 and 7.5 mL) for 2 h. For the second analysis, equal amounts of polymer/Fe_3_O_4_ nanoparticles (20 mg) were exposed to 2.5 mL of gold colloidal solution and the absorbance peak at about 520 nm was measured every 30 min over the course of 2 h.

### Nanoparticle characterization

For the morphology analysis, all particles were subjected to TEM (Zeiss, EM10C, 80 kV, Germany) and DLS analysis (Zetasizer nano-ZS, Malvern, England) for the estimation of the size and surface charge of the produced nanoparticles. XRD (Philips, pw 1800, Netherlands) and EDXD were used for structural and elemental analysis. The XRD method provided significant information about the quantity and quality of the synthesized nanoparticles. To understand the effect of coating on the magnetization behavior of Fe_3_O_4_, the magnetic nanoparticles were subjected to VSM (MAG-3110, Freescale) analysis. By means of FTIR (Thermo Nicolet Nexus 870 FTIR, USA) and using KBr pellets, the assembly of the Au nanoparticles on magnetic nanoparticles was analyzed based on a comparison of the nanoparticles. The FTIR peaks were observed due to the vibrational normal modes, which mostly include stretching vibrations and bending vibrations. The TGA technique (TGA-50, Shimadzu, Japan) was used for the analysis of the thermal stability. Furthermore, from the TGA results, the amount of immobilized polymer on the nanoparticles was estimated. Due to the strong surface plasmon peak of the Au nanoparticles at about 520 nm, the presence of such nanoparticles could be confirmed in the visible spectra via UV–vis absorption spectra (Lambda 950 UV–vis spectrophotometer, Perkin Elmer, USA), verifying the synthesis of the Au nanoparticles.

### Polymer characterization

^1^H NMR spectra were measured in D_2_O at 80 °C using a 300 or 600 MHz spectrometer. ^13^C NMR spectra were measured in D_2_O at 80 °C at 150 MHz. No attempts were made to remove the residual water from the NMR sample because at 80 °C this peak does not interfere with the spectrum of the polymer [39,53]. The ^1^H NMR spectra were used to determine the degree of quaternization (DQ) [39]. Furthermore, both of the polymers were analyzed using FTIR for analysis of inter- and intra-molecular interaction during synthesis of nanoparticles.

### Cell viability assay

The T47D cells were seeded on 96-well plates with 8 × 10^3^ cells in 150 μL RPMI1640 medium per well. The cells were cultured in a medium containing different concentrations (3, 6, and 10%) of FBS and different concentrations of the Au/TMC/Fe_3_O_4_ nanocomposites (20, 50, 80, 110, and 140 µg/mL) for 48 h with a 24 h starvation time. The culture medium without the Au/TMC/Fe_3_O_4_ nanocomposites served as the control in each experiment. At the end of the exposure, 20 μL of MTT reagent was added to each well to reach a final concentration of 2 mg/mL. Afterwards, the cells were further incubated for 3 h at 37 °C. The medium was then cautiously removed and 150 μL of detergent was added and carefully mixed with the cells until formazan crystals were completely dissolved. The plates were read in an ELISA reader at 570 nm. The cell viability was expressed as a percentage of the viability of the control culture [48,54].
